# Editorial: Artificial intelligence advancing lung cancer screening and treatment

**DOI:** 10.3389/fonc.2026.1890285

**Published:** 2026-06-09

**Authors:** Sunyi Zheng, Nanna Maria Sijtsema, Chunliang Wang, Marly Van Assen, Xiaonan Cui

**Affiliations:** 1 Department of Radiology, Medical Artificial General Intelligence for Computation (MAGIC) Lab, National Clinical Research Center for Cancer, Tianjin’s Clinical Research Center for Cancer, State Key Laboratory of Druggability Evaluation and Systematic Translational Medicine, Tianjin Key Laboratory of Digestive Cancer, Tianjin Key Laboratory of Cancer Prevention and Therapy, Tianjin Medical University Cancer Institute and Hospital, Tianjin, China; 2 University of Groningen, University Medical Center Groningen, Department of Radiation Oncology, Groningen, Netherlands; 3 Royal Institute of Technology, Stockholm, Sweden; 4 Department of Radiology and Imaging Sciences, Emory University Hospital, Atlanta, GA, United States

**Keywords:** artificial intelligence, clinical decision support, lung cancer screening, precision medicine, radiomics

Lung cancer remains the leading cause of cancer-related mortality worldwide (1), largely due to late-stage diagnosis. Over the past decade, the implementation of low-dose CT screening has altered the clinical landscape by enabling earlier detection and improved survival outcomes (2, 3). However, this shift has also introduced new challenges, including the interpretation of increasingly large imaging datasets, growing diagnostic complexity, the management of indeterminate pulmonary nodules, and the need for more individualized treatment strategies. In this context, artificial intelligence (AI) has emerged as a potentially important tool across different stages of lung cancer care.

This Research Topic aimed to bring a range of studies that examine the role of AI in lung cancer screening and treatment, spanning early detection, diagnostic characterization, molecular inference, treatment decision-making, and prognostic modeling. Taken together, these contributions reflect a gradual transition from isolated algorithm development toward more clinically oriented and integrative approaches.

## Expanding the scope of lung cancer screening

A recurring theme in this Research Topic is the expansion of lung cancer screening beyond conventional CT-based screening approaches. While CT remains central to current screening strategies (4), several studies explore multimodal approaches that integrate imaging with additional sources of clinical information. One direction involves the incorporation of serum biomarkers for non-invasive risk stratification and early disease assessment (Zhao et al.). Another direction is the use of alternative imaging modalities such as ultrasound, which may provide complementary diagnostic information in selected clinical scenarios (Wei et al.). These developments indicate that lung cancer screening may increasingly evolve toward a multimodal framework.

## From visual interpretation to quantitative phenotyping

Beyond nodule detection, several studies focus on improving diagnostic accuracy through quantitative imaging analysis. Radiomics-based models incorporating both intranodular and perinodular features demonstrate promising performance in differentiating pulmonary cryptococcosis from lung adenocarcinoma (Deng et al.), highlighting the potential value of capturing information from both the tumor and its surrounding environment. Similarly, models combining radiomic features and clinical-semantics show promising performance in distinguishing histological subtypes of non-small cell lung cancer, particularly squamous cell carcinoma and adenocarcinoma (Li et al.). These approaches suggest that quantitative imaging analysis may provide complementary information beyond conventional visual assessment. By extracting high-dimensional features, AI-based methods may identify subtle imaging patterns that are difficult to appreciate visually, potentially supporting more accurate diagnostic characterization and guide further follow-up decisions.

## Bridging imaging and tumor biology

Another area of interest is the application of AI to link imaging findings with underlying tumor biology. Several studies report that radiomic and deep learning models can predict molecular characteristics, including gene expression patterns and mutation status (Li et al. and Luo et al.). The integration of radiomics with single-cell transcriptomic data also represents an emerging direction, enabling the linkage of imaging features with molecular phenotypes and metastasis-associated gene expression (Wu et al.). In addition, deep learning applied to histopathological images has been used to identify morphological patterns associated with treatment response, extending beyond conventional biomarkers such as PD-L1 expression (Peroz et al.). These findings show that AI-based approaches may contribute to noninvasive characterization of tumor biology, although further validation is required before clinical implementation.

## Toward AI-informed treatment decision-making

AI applications are also being explored in the context of treatment decision support. Models predicting immunotherapy response and immune-related adverse events illustrate the potential for integrating clinical and imaging features to support therapeutic planning (Cao et al. and Hou et al.). Such approaches may help to balance treatment efficacy and toxicity at an individual level. Furthermore, models trained on multidisciplinary tumor board decisions show that machine learning can approximate complex clinical decision processes (Pasello et al.). While these approaches are still at an early stage, they may have potential in supporting decision-making in settings with limited access to expert consensus.

## Extending AI across the patient trajectory

In addition to diagnosis and treatment selection, this Research Topic shows that AI is being applied to prognostic modeling and perioperative risk assessment. Studies predicting postoperative recurrence (Lan et al.) and pulmonary complications suggest that AI may support longitudinal patient management (Sha et al.). Early identification of high-risk patients could facilitate more tailored follow-up and intervention strategies. These applications indicate that AI may support multiple stages of the clinical workflow, extending beyond isolated tasks toward more continuous and longitudinal patient management.

## Methodological trends and emerging challenges

Methodological trends can be observed across the studies included in this Research Topic. First, there is increasing use of multimodal data integration, combining imaging, clinical, and molecular information. Such approaches may provide more comprehensive characterization of tumor biology and support more individualized clinical assessment. Second, model interpretability is receiving growing attention, with approaches such as SHAP and Grad-CAM used to provide insights into model decision-making (5, 6). Improved interpretability may facilitate clinical understanding and increase confidence in AI-assisted decision-making. Third, increasing attention is being paid to external validation and generalizability, with multi-center datasets being used more frequently. Despite these advances, several challenges remain, including data heterogeneity, limited standardization of radiomics workflows, and variability in methodological and reporting quality, which may affect reproducibility and broader clinical translation.

## Future directions

Future work should focus on improving the clinical applicability of AI-based approaches in lung cancer screening and treatment. In particular, further efforts toward standardization of data processing workflows and reporting practices will be important to improve reproducibility and comparability across studies (7). More appropriate evaluation strategies may also be needed in certain clinical scenarios, such as the use of precision-recall metrics alongside conventional AUC-based assessment for imbalanced datasets. In parallel, broader integration of multimodal data, including imaging, pathology, molecular, and longitudinal clinical information, may support more comprehensive characterization of tumor biology and patient status. Finally, prospective studies and real-world evaluations will remain important to assess the clinical utility, workflow integration, and practical implementation of AI-based tools.

## Conclusion

The studies included in this Research Topic provide an overview of current applications of artificial intelligence in lung cancer screening and treatment. Across different stages of care, AI-based approaches show potential to complement existing methods and support clinical decision-making. While further validation and standardization are needed, these developments suggest a gradual movement toward more data-driven and individualized approaches in lung cancer management.
